# Loss of Hepatocyte-Nuclear-Factor-4α Affects Colonic Ion Transport and Causes Chronic Inflammation Resembling Inflammatory Bowel Disease in Mice

**DOI:** 10.1371/journal.pone.0007609

**Published:** 2009-10-29

**Authors:** Mathieu Darsigny, Jean-Philippe Babeu, Andrée-Anne Dupuis, Emma E. Furth, Ernest G. Seidman, Émile Lévy, Elena F. Verdu, Fernand-Pierre Gendron, François Boudreau

**Affiliations:** 1 Canadian Institutes of Health Research Team on Digestive Epithelium, Département d'anatomie et biologie cellulaire, Faculté de Médecine et des Sciences de la Santé, Université de Sherbrooke, Sherbrooke, Quebec, Canada; 2 Département d'anatomie et biologie cellulaire, Faculté de Médecine et des Sciences de la Santé, Université de Sherbrooke, Sherbrooke, Quebec, Canada; 3 Department of Pathology and Laboratory Medicine, University of Pennsylvania, Philadelphia, Pennsylvania, United States of America; 4 Research Institute, McGill University Health Center, Montréal, Quebec, Canada; 5 Department of Nutrition, CHU Ste-Justine, Université de Montréal, Quebec, Canada; 6 Division of Gastroenterology, McMaster University, Hamilton, Ontario, Canada; Charité-Universitätsmedizin Berlin, Germany

## Abstract

**Background:**

Hnf4α, an epithelial specific transcriptional regulator, is decreased in inflammatory bowel disease and protects against chemically-induced colitis in mice. However, the precise role of this factor in maintaining normal inflammatory homeostasis of the intestine remains unclear. The aim of this study was to evaluate the sole role of epithelial Hnf4α in the maintenance of gut inflammatory homeostasis in mice.

**Methodology/Principal Findings:**

We show here that specific epithelial deletion of *Hnf4α* in mice causes spontaneous chronic intestinal inflammation leading to focal areas of crypt dropout, increased cytokines and chemokines secretion, immune cell infiltrates and crypt hyperplasia. A gene profiling analysis in diseased *Hnf4α* null colon confirms profound genetic changes in cell death and proliferative behaviour related to cancer. Among the genes involved in the immune protection through epithelial barrier function, we identify the ion transporter claudin-15 to be down-modulated early in the colon of *Hnf4α* mutants. This coincides with a significant decrease of mucosal ion transport but not of barrier permeability in young animals prior to the manifestation of the disease. We confirm that claudin-15 is a direct Hnf4α gene target in the intestinal epithelial context and is down-modulated in mouse experimental colitis and inflammatory bowel disease.

**Conclusion:**

Our results highlight the critical role of Hnf4α to maintain intestinal inflammatory homeostasis during mouse adult life and uncover a novel function for Hnf4α in the regulation of claudin-15 expression. This establishes Hnf4α as a mediator of ion epithelial transport, an important process for the maintenance of gut inflammatory homeostasis.

## Introduction

Hepatocyte-nuclear-factor-4α (Hnf4α) was originally identified as an endoderm specific transcriptional regulator detectable in the liver, pancreas and intestine, essential for embryonic development [1]. Conditional genetic removal of *Hnf4α* function in the liver results in impaired lipid metabolism and gluconeogenesis [2] and disorganization of morphological and functional differentiation in the hepatic epithelium [3]. One critical set of gene products found to be disrupted in hepatocytes that have lost Hnf4α is related to epithelial cell adhesion and junction formation [4]. Thus, Hnf4α represents a potent transcriptional regulator with a strong impact on endodermal development and metabolism related pathophysiology.

HNF4α has recently emerged as being a potential regulator of intestinal epithelial function. *In vitro* studies suggest that it stimulates intestinal epithelial cell differentiation, resulting in the formation of a tight epithelial barrier [5]. These features are central to the differentiated epithelium to ensure nutrient metabolism [6] and barrier protection against pathogens [7]. Another fundamental characteristic of the epithelial barrier is to regulate appropriate ion selectivity, for which impairment contributes to the manifestation of inflammatory bowel disease (IBD) [8], [9]. Hnf4α is important during early embryonic colon development and regulation of genes related to epithelial functions [10]. The intestinal epithelial role of Hnf4α is of less importance when disrupted during post-embryonic development of the gut [11]. Hnf4α is required to protect the epithelium during experimental colitis in young adult mice and is reduced in IBD [12]. However, no inflammatory defect was reported to occur in animals that were not chemically induced to develop colitis leaving questionable whether Hnf4α was functionally important to maintain appropriate inflammatory homeostasis during adult life.

Herein, we aimed to investigate whether epithelial *Hnf4α* was solely involved in the control of intestinal inflammatory homeostasis and to explore the nature of the mechanisms implicated. Our results show that *Hnf4α* deletion spontaneously triggered chronic inflammatory response in the colon characterized by disrupted crypt architecture, proliferative changes, apoptosis, increased inflammatory mediator secretion and increased immune cell infiltration. We propose that the loss of mucosal homeostasis is initiated by an early reduction of mucosal ion transport, which was not associated with a significant change of barrier permeability. Claudin-15, a paracellular ion transporter, was confirmed to be a novel and direct gene target for Hnf4α. These findings identify Hnf4α as a transcriptional regulator of ion transport and support a novel functional role for this transcriptional regulator in colonic inflammatory homeostasis.

## Materials and Methods

### Animals and Ethics Statement

12.4 KbVilCre/*Hnf4α^loxP-loxP^* mutant mice were obtained exactly as described previously [11]. Mice were kept under pathogen free conditions and were tested negative for helicobacter, pasteurella and murine norovirus. Mice were treated in accordance with a protocol approved by the Institutional Animal Research Review Committee of the Université de Sherbrooke.

### RNA Isolation and Quantitative PCR

Tissue samples were harvested, total RNA was isolated and quantitative RT-PCR was performed as described previously [13]. Target expressions were normalized using either hypoxanthine-guanine phosphoribosyl transferase (HPRT) or TATA binding protein (TBP) expression depending on target mRNA abundance. All primer sequences and cycling conditions are available upon request.

### Microarray Screening and Data Analysis

Probes for microarray analysis were generated from RNA isolated from the proximal colon of 3 control and 3 *Hnf4α* mutant mice at 12 months of age. Affymetrix GeneChip® Mouse Genome 430 2.0 arrays were screened with six generated probes via the microarray platform of McGill University and Génome Québec Innovation Center as previously described [14] (data are accessible through GEO series accession number GSE11759). To test for statistically significant changes in signal intensity (*p* values of≤0.05; SAM test), compiled data (RMA analysis) were screened using FlexArray 1.1 (Genome Quebec). Data were also analyzed through the use of Ingenuity Pathways Analysis (IPA) to generate networks of genes associated with biological functions and/or diseases (Ingenuity Systems, www.ingenuity.com).

### Immunoblots

Total protein extracts were obtained and separated by protein electrophoresis as described previously [13]. The following antibodies were used and incubated at 4°C O/N: anti-β-actin (#sc1615, 10 ng/ml) and anti-HNF4α (#sc6556, 100 ng/ml) from Santa Cruz; anti-cleaved caspase-3 (#9664, 1∶500), anti-IκBα (#9242, 1∶1000), anti-phospho- IκBα (Ser32) (#2859, 1∶1000) from Cell Signalling and anti-claudin-4 (#36-4800, 500 ng/ml), anti-claudin-8 (#40-0700Z, 32 ng/ml), anti-claudin-15 (#38-9200, 250 ng/ml) and anti-E-cadherin (#33-400, 30 ng/ml) from Zymed.

### Electronic Microscopy

Tissues intended for electronic microscopy analysis were fixed with 2.5% glutaraldehyde in 0.1 M cacodylate buffer and processed as described previously [13].

### Immunohistochemistry, Immunofluorescence and Cytological Stains

For histological analysis, tissues were fixed in a swiss roll position using 10% neutral buffered formalin and paraffin embedded. Antigen retrieval was performed using 10 mM citrate buffer, pH 6.0 and the DAKO EnVision+ System-HRP according to the manufacturer's instructions (DakoCytomation). Antibodies were used under the following conditions: anti-MPO (#RB-373, 1∶100) and anti-Ki67 (#RB-1510-R7) (Neomarkers). For HNF4α indirect immunofluorescence, anti-HNF4α (1 mg/ml) was used. For claudin immunodetection, 5 µm thick OCT cryosections were fixed in 100% methanol for 10 minutes at – 20°C followed by incubation with anti-claudin-4 (1 µg/ml), anti-claudin-8 (0.2 µg/ml) and anti-claudin-15 (1 µg/ml). Primary antibodies were visualized with the appropriate FITC-coupled secondary antibody (Vector Laboratories) and samples were mounted with Vectashield Hard Set mounting medium with DAPI (Vector Laboratories). Alcian blue staining was done as described elsewhere [15]. All images were captured on a Leica DMLB2 microscope using a Leica DFC300 FX camera.

### Cytokine Array and ELISA

Cytokine production by colonic tissues of normal and Hnf4α null mice was determined using the RayBio® Mouse Cytokine Antibody Array G series 1000 (RayBiotech, Norcross, GA). Colons of 1 year old mice were harvested and protein lysates prepared as previously described [16]. The cytokine antibody array was performed with 40 µg of protein and scanned in the Cy3 channel using a ScanArray Express dual-color confocal laser scanner (Perkin Elmer). Results were normalized and analyzed as recommended by the manufacturer [17]. CXCL1 expression was quantified using the R&D Systems duoSet ELISA development kit for mouse CXC chemokine KC (R&D Systems). ELISA assays were performed using 30 µg of whole colon protein extract per well following the manufacturer's recommendation.

### DSS Samples and UC Samples

2.5% DSS mild treatment was given to CD1 mice for a period of 10 days and the colon was harvested. TissueScan Real-Time colitis disease panels were purchased from Origene Technologies. Each panel was composed of total isolated RNA from 6 control samples and 21 UC samples collected all from different individuals. Lyophilised cDNAs were resuspended and qRT-PCR was performed as described above and normalized against β-actin. PCR conditions and primers used are available upon request.

### EMSA and Chromatin Immunoprecipitation

EMSA were performed exactly as described previously [18]. For chromatin immunoprecipitation, whole colon was removed, fixed in 1% formaldehyde and the epithelial cells harvested with a cell recovery solution (BD bioscience). Cells were then sonicated and ChIP performed with Ez-Chip as described by the manufacturer (Millipore).

### Cell Culture

Full length claudin-15 (*NM_021719.3, 1..1846*) was amplified from mouse cDNA with iproof polymerase (Bio-Rad) and cloned in plenti6/V5 vector. Lentiviruses were produced and infection of T84 and IEC6 epithelial cell lines (obtained from the American Type Culture Collection, catalog no. CCL-248 and CRL-1592) was performed as described previously [13]. Stable T84 cell populations were seeded on Corning Transwell permeable supports and electrical resistance was measured each day following the reach of confluency with EVOM instrument (World Precision Instrument).

### Ion Transport and Permeability Measurement

Two sections of colon from each mouse were used for epithelial ion transport and permeability measurements [19]. Each colonic segment was opened along the mesenteric border, rinsed, and mounted in an Ussing chamber. Tissues were bathed in oxygenated Krebs buffer containing 10 mM glucose (serosal side) or 10 nM mannitol (luminal side) at 37°C, and net active transport across the epithelium was measured via a short circuit current (*I*_sc_; µA) injected through the tissue under voltage-clamp conditions. After a 15-min equilibration period, baseline *I*_sc_ (µA/cm^2^) was recorded. Colon paracellular permeability was measured using recovery of 51-chromium-ethylenediaminetetraacetic acid (^51^Cr-EDTA). Briefly, 6 µCi of ^51^Cr-EDTA/ml was added to the luminal side of tissues. 100 µl samples were taken from the serosal side at 0, 30, 60, 90 and 120 min and compared to 100 µl of the radioactive sample (100%) obtained from the luminal side at time 0 [20].

### Statistical Analyses

All data were expressed as mean ± SEM. Groups were compared with student *t* test unless otherwise noted (GraphPad Prism 5, GraphPad Software, SD). Statistical significance was defined as *P*<.05.

## Results

### Intestinal Epithelial Specific Deletion of Hnf4α Alters Adult Colonic Crypt Integrity and Result in Spontaneous Colonic Inflammation

**Figure 1:**
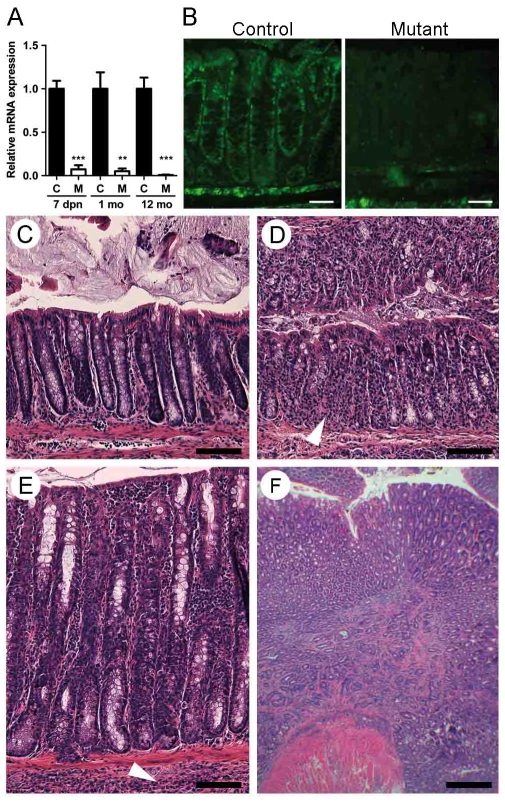
Epithelial specific loss of Hnf4α leads to various signs of chronic colon inflammation. (A) qRT-PCR of *Hnf4α* mRNA at 7 days, 1 month and 12 months in mutants (open bars) relative to controls (black bars). Expression levels are shown as mean values (±SEM) relative to controls for each time point (3–8 mice per group) and were normalized with the *Tbp* housekeeping gene. **P≤.01 and ***P≤.001. (B) Indirect immunofluorescence of Hnf4α performed on controls and *Hnf4α* colon null mice. A non-specific fluorescent signal was observed in the stroma of both control and null mice. Scale bars, 50 µm. (C) H&E staining of a healthy mucosa in mutants. (D) Mucosa with signs of crypt distortion and crypt loss. (E) Increased crypt length and cell infiltrates. (F) Polyp formation. These photographs are representative of eight 12 month-old individuals all showing these morphological features. The formation of polyps was only observed in 2 independent *Hnf4α* colon null mice. All scale bars are 100 µm except for panel F that is 200 µm.

**Figure 2:**
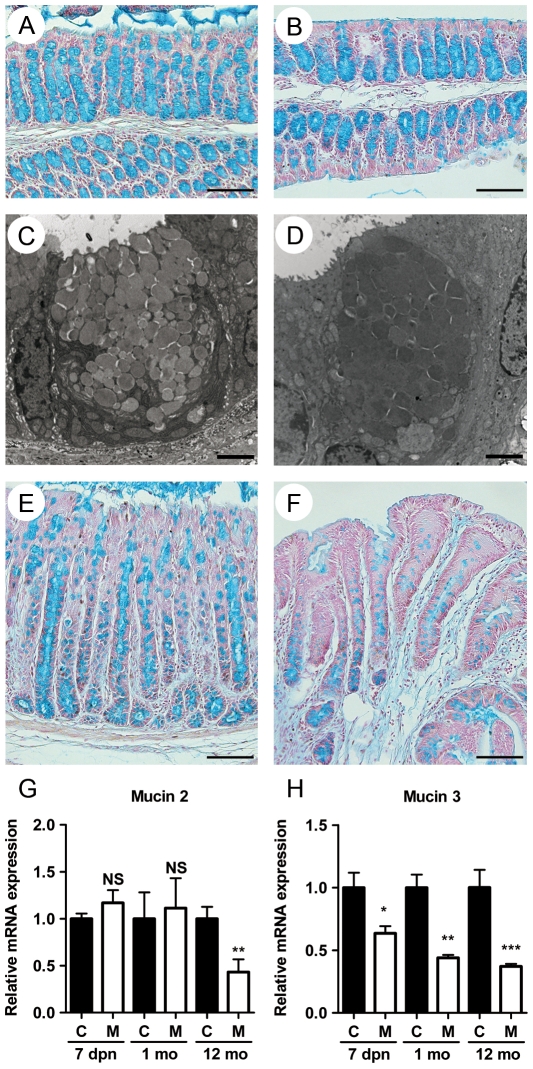
Loss of Hnf4α does not compromise goblet cell maturation in healthy colon mucosa but in long term inflamed colon mucosa. (A) Alcian blue staining of control mice aged of 12 months. (B) Healthy colon region of 12 month-old mutant mice showing normal size and number of goblet cells (Scale bar, 100 µm). Electron microscopy shows a similar maturation level of goblet cells in control (C) and mutant mice (D) (Scale bar, 2 µm). (E) Small size goblet cells in hyperplasic epithelium of 12 month-old mutant mice. (F) Drastic reduction in goblet cell number in hyperplasic crypts (Scale bar, 100 µm). (G) qRT-PCR of *Muc2* mRNA and (H) *Muc3* mRNA in Hnf4α mutants (Open bars) and controls (Black bars). Expression levels are shown as mean values (± SEM) relative to controls and were normalized with the *Hprt* gene. **P≤0.01 and ***P≤0.001.

**Figure 3:**
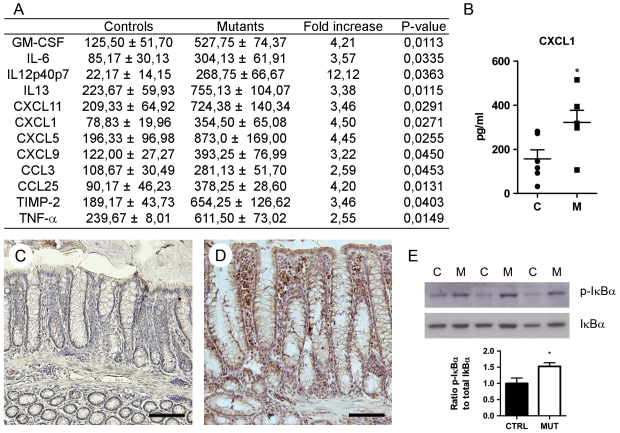
Increased cytokine and chemokine secretion associated with the inflammatory state of *Hnf4α* colon null mice. (A) Summarized results (means ± SEM; at least 3 individuals aged of 12 months) of the semi-quantitative cytokine/chemokine array shown in Table S1. (B) ELISA performed on total protein lysates (means ± SEM; n = 6 per group). (C) Labelling of granulocytic cells by MPO immunohistochemistry was negative in controls as opposed to (D) mutants. Scale bars, 100 µm. (E) Western blot of accumulated phospho-IκBα relative to total IκBα and relative quantification (mean ± SEM), *P≤.05.

A mouse *Hnf4α* intestinal epithelial conditional knockout colony was generated exactly as described recently [11]. Total RNA isolated from the colon and subjected to qRT-PCR showed that mutant mice did not produce significant levels of wild-type *Hnf4α* mRNA (Figure 1A). Indirect immunofluorescence confirmed that production of the Hnf4α protein was abolished in the colon of mutant mice as compared to control mice (Figure 1B). Intestinal morphology was then compared in tissue sections prepared from control and mutant mice. No significant alteration of the intestinal mucosa was observed in young adult mice. Mild colonic crypt distortion was detected in 3 month old mutant mice. This phenotype worsened over time, becoming severe and fully penetrant for each 6 to 12 months old mutant mouse analysed (Figure 1). Histological analysis of colon sections from these mice revealed healthy crypt regions (Figure 1C) surrounded with abundant focal regions of colonic mucosa with signs of acute inflammation and epithelial destruction (Figure 1D), crypt hyperplasia associated with increased submucosal immune cell infiltrates (Figure 1E) and in rare occasion, early signs of neoplasia that was never observed in age-matched control mice (Figure 1F). Goblet cells in the colonic mucosa were stained with Alcian blue and no significant differences were observed between control (Figure 2A) and non-inflamed regions of the adult mutant mice (Figure 2B). Electron microscopy confirmed the similarities in goblet cell maturity between control (Figure 2C) and mutant mice (Figure 2D). However, the size of goblet cells was reduced in enlarged crypts (Figure 2E) and decreased in number in the more severely distorted crypts of the *Hnf4α* mutant mice (Figure 2F). Relative mRNA levels of *Muc2*, a specific marker of goblet cells, remained stable during the first 3 months but significantly declined in older *Hnf4α* mutant animals (Figure 2G). The expression of the *Muc3* gene decreased early during post-natal life (Figure 2H). Consistent with the similarity to the histopathology seen in patients with IBD, some of the *Hnf4α* mutant mice displayed a characteristic phenotype with relapsing episodes of hematochezia and rectal prolapse (data not illustrated). To further assess the expression of modulators of the immune response, a cytokine antibody array assay was performed on *Hnf4α* colon null and control total protein extracts (Table S1). This analysis identified several cytokines and chemokines to be significantly up-regulated in the *Hnf4α* colon mutant mice (Figure 3A). An ELISA confirmed that the epithelial chemokine C-X-C ligand 1 (CXCL1) was significantly elevated in these samples (Figure 3B). This up-regulation was further correlated to the increased detection of the myeloperoxidase (MPO) signal associated with leukocytes that are recruited following the chemoattractant properties of CXCL1 released at inflammation site (Figure 3C, D). NFκB activity was significantly increased in *Hnf4α* colon mutant mice as determined by the level of phosphorylated IκBα (Figure 3E). Overall, these observations demonstrated that the loss of Hnf4α results in spontaneous chronic inflammation resembling inflammatory bowel disease.

### Expression of Cell Death, Cancer and Gastrointestinal Diseases Related Genes Is Altered in the Colon of Hnf4α Mutant Mice

Gene expression profiling was performed to clarify the gene network associated with the loss of *Hnf4α* and the long term morphological changes observed in colonic crypts. A statistical analysis (*p*≤.05) predicted 485 unique mouse transcripts to be modulated between control and mutant animals (differential ratio ≥2.0, Table S2). To gain insight into how these modifications relate to colonic function, IPA software was utilized to categorize these modified genes by biological function and to extrapolate interaction networks among them. This analysis identified cell death and cell cycle as being the most important cellular processes predicted to be affected in the *Hnf4α* null colon (Figure S1). Of interest, the most significant diseases and disorders that were predicted to occur based on modifications in gene transcripts expression was cancer and gastrointestinal diseases.

**Figure 4:**
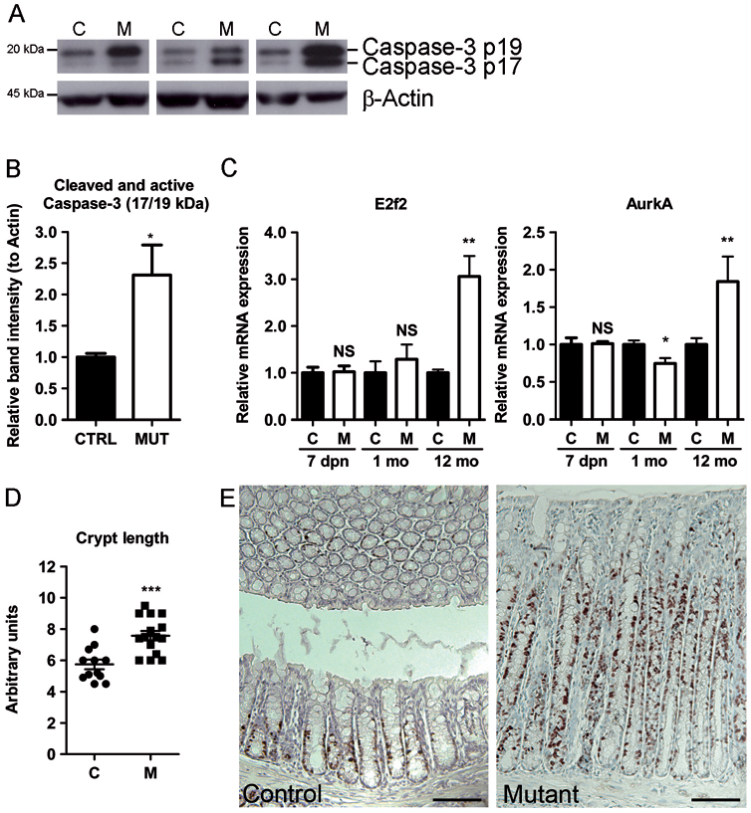
The apoptotic pathway is overly active and the proliferative dynamic is altered in regenerative epithelium. (A) Increased abundance of cleaved and active forms of caspase-3 in 12 month-old mutants (Open bar) relative to controls (Black bar). (B) Quantification of band intensities of cleaved and active caspase-3 combined (n = 4) normalized to β-actin (Black bars, controls; open bars, mutants). Represented as means ± SEM, * *P*≤.05. (C) *E2f2* and *AurkA* modulations seen in microarray data were verified at various time points in mutants (Open bars) relative to controls (Black bars). Expression levels are shown as mean values (± SEM) relative to controls for each time point (3–14 mice per group) and were normalized with the *Tbp* housekeeping gene. (D) Measurements (n = 4 per group) of crypt length. (E) Ki67 immunochemistry of 12 month-old control mice compared to a hyperplasic area seen in mutants of same age. * *P*≤.05, ** *P*≤.01 and *** *P*≤.001.

Identification of these specific molecular-physiological networks led us to show that specific cleavage of caspase-3, a critical executioner during cell apoptosis, was significantly increased in the colon of mutant *Hnf4α* mice (Figure 4A and B). However, the cleavage of caspase-3 was not significantly altered in young adult mice (data not shown). The expression of the *E2f2* transcript, a marker for both cell proliferation and apoptosis [21], remained stable in early postnatal life but became significantly increased in the colon of 12 months old mice (Figure 4C). The expression of the aurora kinase A (*AurkA*) transcript, a regulator of mitosis that is up-regulated in patients with ulcerative colitis [22], was also significantly up-regulated in the long term in colon of *Hnf4α* mutant mice (Figure 4C). These observations were consistent with the long term significant increase in colon crypt length observed in 12 months old *Hnf4α* mutant mice (Figure 4D) and the drastic increase in Ki67 labeling in elongated crypts of *Hnf4α* colon null mice as compared to controls (Figure 4E). Thus, apoptosis and cell proliferation was increased in the long term in colon disease of *Hnf4α* mutant mice probably as a consequence to the inflammatory response.

### Ion Channel/Transporters Are Modulated Early in Hnf4α Colon Mutant Mice

**Figure 5:**
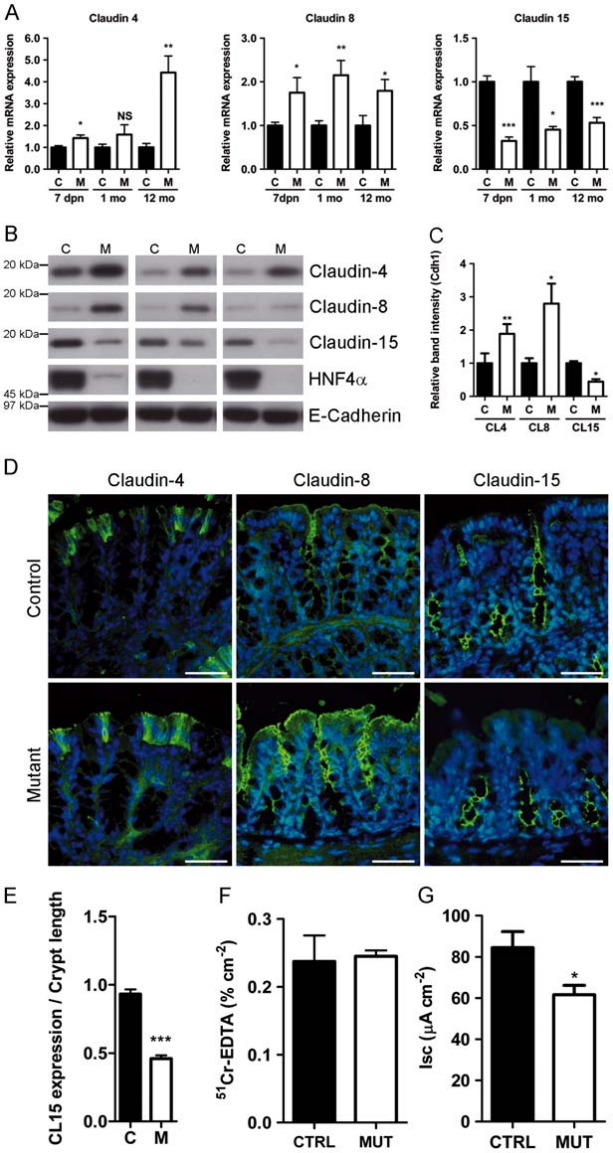
Claudin-15 and ion transport are modulated in *Hnf4α* colon null mutants. (A) qRT-PCR of claudin-4, -8 and -15 at various time points in mutants (Open bars) relative to controls (Black bars). Expression levels are shown as mean values (± SEM) relative to controls at each time point (3–14 mice per group) and were normalized with the *Tbp* housekeeping gene. (B) Immunoblotting confirmed modulated protein levels of claudin-4, -8 and -15 at 12 months in mutants (n = 3, open bars) and relative controls (n = 3, black bars) normalized to stable expression of E-Cadherin (Cdh1). (C) Quantification of band intensities of each claudin (means ± SEM; n = 3 or 4 for each group) relative to Cdh1. (D) Indirect immunofluorescence (FITC) of claudin-4, -8 and -15 in 3 month-old animals in proximal colon (representative of 4 pairs). Nuclei were counterstained with DAPI (blue) and merged with FITC (green). Scale bars, 50 µm. (E) Claudin-15 expression is lost in the upper half of the colon epithelium in mutants (Open bar) relative to controls (Black bar). (F) Colonic permeability to ^51^Cr-EDTA measured in colon tissues from 2 month-old mice (5 mice per group). (G) Baseline short circuit current (Isc) measured in colon tissues from 2 month-old mice (5 mice per group). Represented as mean values ± SEM, * *P*≤.05, ** *P*≤.01, *** *P*≤.001.

Hnf4α induces epithelial polarity and barrier function in intestinal epithelial cells *in vitro* [5] and is a crucial regulator of multiple genes involved in tight and adherent junctions in hepatocytes [4]. We thus looked for transcript variation in components of cell-to-cell junctional complexes to monitor whether loss of Hnf4α could affect these transcriptional targets. Surprisingly, this analysis identified very few modifications in the expression of these classes of genes except for the down modulation of claudin-15 transcript and a probable indirect induction of claudin-4 and -8 gene transcripts (Table S3). Gene transcript quantification for each of these claudins confirmed an induction of claudin-4 and claudin-8 and a reduction of claudin-15 in *Hnf4α* colon null mice (Figure 5A). These changes were observed as early as postnatal day 7, a period that preceded detection of crypt distortion and inflammation. Western blot analysis confirmed that these variations were of consequence on the respective protein level in the colon of adult mice (Figure 5B, C). Immunolocalization of both claudin-4 and claudin-8 showed a stronger signal on the colon epithelial surface of *Hnf4α* colon null mice as compared to controls (Figure 5D). Although claudin-15 protein was localized throughout the colonic crypt in control mice, its distribution changed drastically in the *Hnf4α* colon null mice with the upper half of the crypt being devoid of claudin-15 (Figure 5D and E). Because claudin-15 was reported to be crucial in maintaining ion transport but not mucosal permeability in the gut [23], [24], mucosal barrier assays were next performed. Young adult mutant mice did not show significant alteration in colon mucosal permeability to ^51^Cr-EDTA as compared to controls (Figure 5F). This observation coincided with the fact that a limited subset of tight and adherens junction related genes were modulated in the *Hnf4α* colon null mice (Table S3). However, baseline *I*_sc_ was significantly decreased in the colon of young adult *Hnf4α* null mice as compared to controls demonstrating that a decrease in active epithelial ion transport in absence of Hnf4α was taking place before the onset of the disease (Figure 5G). In addition to claudin-15 being crucial for ion transport function, more than 39 gene transcripts directly associated with ion transport function were predicted to be significantly reduced in diseased *Hnf4α* colon null mice (Table S4).

### Claudin-15 Is a Direct Gene Target for Hnf4α and Increases Paracellular Conductance in Polarized T84 Colonic Epithelial Cells

**Figure 6:**
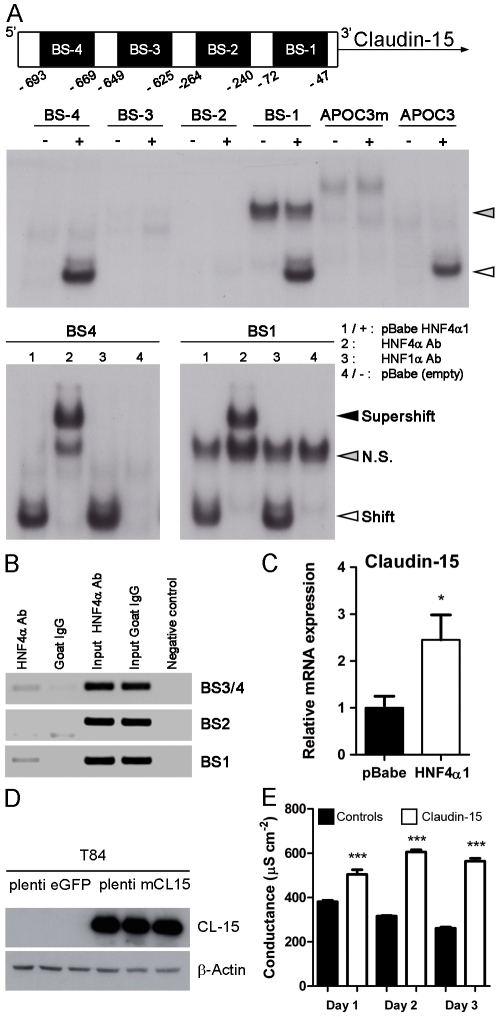
The claudin-15 gene is a direct target of Hnf4α. (A) EMSA of 4 Hnf4α binding sites found in the 1 kb mouse claudin-15 promoter performed with 293T cells transfected with pBabe Hnf4α1 vector and 293T nuclear extracts as negative controls. Shifts (white arrow head) are compared to previously published APOC3 and mutated APOC3 (APOC3m) for control binding site. NS is non-specific binding (grey arrow head). Supershifts (black arrow head) were done with Hnf4α antibody (sc-6556). (B) ChIP assays of the 4 putative sites performed on epithelial cells from adult mouse colon. (C) qRT-PCR of claudin-15 on IEC6 cells infected with pBabe Hnf4α as compared to control vector pBabe (n = 4). (D) Western blot of 3 stable T84 cell populations expressing mouse claudin-15. (E) Conductance measured for three consecutive days after the reach of confluence on three populations of T84 cells expressing mouse claudin-15 or control plenti eGFP vector. Represented as mean values±SEM, *** *P*≤.001.

Since Claudin-15 (Cldn15) was one of the earliest modified transcripts in *Hnf4α* colon null mice, we verified its status as a direct gene target for Hnf4α. Analysis of the first 1 kb of the mouse *Cldn15* gene promoter with the TRANSFAC database predicted 4 putative Hnf4α binding sites (Figure 6A). The ability of Hnf4α to interact with these sites was monitored by EMSA. Nuclear extracts from 293T cells expressing exogenous Hnf4α led to a specific shift complex for two of the predicted sites (BS-1 and BS-4) in a comparable manner to a previously validated Hnf4α site within the *Apoc3* gene promoter [10] (Figure 6A). This shift complex was specific for Hnf4α interaction as demonstrated with the formation of a supershift complex when Hnf4α antibodies were included in the binding reaction but not when substituted with control antibodies (Figure 6A). ChIP assays were next performed on >isolated mouse colonocytes. Immunoprecipitations of the chromatin with Hnf4α antibodies followed by PCR amplifications of different *Cldn15* regions were performed. Hnf4α consistently pulled down the BS-1 and BS-4 containing genomic regions of *Cldn15* in contrast to the BS-2 region, negative for Hnf4α interaction by EMSA and not immunoprecipitated under these conditions (Figure 6B). Forced expression of Hnf4α in cultured intestinal epithelial cells resulted in a significant increase of *Cldn15* gene transcript (Figure 6C). Exogenous expression of Cldn15 in the T84 colon cell line was next achieved to monitor the function of Cldn15 on ion conductance in the context of colonic epithelial cells (Figure 6D). Controls and Cldn15 T84 cell monolayers were seeded onto semipermeable filters and conductance was monitored after each cell populations had reached confluence. A significant increase in ion conductance was maintained for several days in T84/Cldn15 as compared to T84 control cell populations (Figure 6E). These results indicated that Hnf4α was able to activate *Cldn15* expression probably via direct transcriptional mechanisms and confirmed that Cldn15 was potent to increase ion conductance in the context of a colonic epithelial cell monolayer.

### Down-Modulation of *HNF4A* Correlates with Decreased Expression of *CLDN15* in Experimental Colitis and IBD

**Figure 7:**
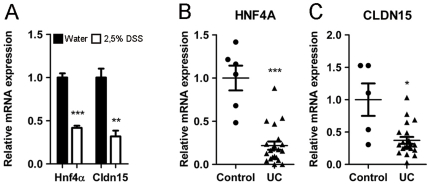
*HNF4A* expression is reduced in IBD patient samples in association with Claudin-15. (A) qRT-PCR of Hnf4α and claudin-15 expression following 10 days of 2.5% DSS mild treatment in the distal colon of CD1 mice relative to water treated mice. *HNF4A* expression was retained at 22% (B) and claudin-15 was retained at 37% (C) in ulcerative colitis (UC) patient samples (n = 21) as compared to healthy patient samples (CTRL) (n = 6). Represented as mean values ± SEM, * *P*≤.05, ** *P*≤.01, *** *P*≤.001.

HNF4α was reported to be decreased during experimental colitis and IBD [12]. The expression status of *CLDN15* in the disease was assessed by qRT-PCR in comparison to *HNF4A*. DSS-induced colitis led to a significant *Cldn15* reduction in the colon similarly to *Hnf4α* levels (Figure 7A). The expression of both *HNF4A* (Figure 7B) and *CLDN15* (Figure 7C) gene transcripts were significantly reduced in intestinal biopsies from IBD patients as compared to controls. These observations further supported a close relationship between *Hnf4α* and *Cldn15* expression during intestinal inflammatory diseases.

## Discussion

HNF4α transcriptional regulator is critical during the ontogeny and normal development of certain organs as well as in the pathogenesis of human disorders. Its roles have been ascertained for homeostasis of liver and pancreatic function and have been functionally linked to diabetes [25]. HNF4α is crucial to the polarization of cultured intestinal epithelial cells and to the initiation of their morphological and genetic differentiation *in vitro* [5]. Hnf4α is also essential to colonic epithelial cell differentiation during early mouse embryonic development [10]. However, small intestine deletion of Hnf4α following embryonic development results in minor defects on epithelial differentiation [11]. Here, our findings identify HNF4α as an important regulator of gut mucosal ion transport rather than mucosal permeability. In addition, a long term and chronic inflammatory consequence arising from the colonic epithelial loss of this transcriptional regulator was observed. The delayed manifestation of the clinical symptoms related to the colonic disease indicates that loss of Hnf4α involves complex mechanistically interactions that ultimately lead to the development of the disease. The pathogenesis of IBD is complex and involves susceptibility genes, the epithelial barrier function, innate and adaptive immunity and environmental factors including bacteria [26]. The only premature defect we were able to identify in the colon of *Hnf4α* null mice was a decline in ion transport. We propose that rapid down-modulation of Hnf4α-dependent transcriptional targets such as *Cldn15* can cause defects in epithelial ion transport ultimately leading to histopathological changes resembling those seen in patients with IBD.

Hnf4α was recently reported to be required in colon epithelial cells to protect against dextran sodium sulphate (DSS), a commonly used model of acute intestinal injury that does not result in chronic inflammatory disease [12]. The *Muc3* gene, a transcriptional target of Hnf4α [10], was reported to be down-modulated in young adult *Hnf4α* colon null mice and was proposed to account for the Hnf4α protective effect. This specific transmembrane mucin is localized both in absorptive and goblet cells [27] and can reduce mucosal ulceration and apoptosis in experimental acute colitis [28]. Thus reduction in Muc3 could be partly involved in the long term spontaneous disease of *Hnf4α* colon null mice. However and in contrast to Ahn *et al*. [12], we observed that goblet cell maturation was not significantly altered in young adult *Hnf4α* colon mutant mice and that the Muc2 gene transcript was only decreased in later stages, in association with the depletion of goblet cells in hyperplasic crypts. The results suggest the latter is not a primary event in the initiation of the disease but rather a consequence of progression of colonic inflammation. Colonic goblet cell maturation was reported to be altered in embryonic *Hnf4α* colon mutant mice with the use of another mouse conditional knockout system [10]. The precise window of time in which intestinal *Hnf4α* was altered differs in our study, as compared to what had been previously reported [10]. The *Foxa3*-Cre transgene utilized in the latter study efficiently removed *Hnf4α* at embryonic day 7.0, a period that precedes the onset of intestinal epithelial monolayer formation and cytodifferentiation. The *Villin*-Cre transgene has been reported to be active at a period following the onset of intestinal cytodifferentiation [11]. We conclude that the presence of Hnf4α no longer interferes with cell epithelial differentiation or goblet cell maturation after determination has occurred during early intestinal ontogeny. Increased mucosal permeability was also reported to occur in adult *Hnf4α* colon null mice when administrated with DSS for several days [12]. Although the exact mechanism by which DSS induces acute colitis are still not well described, there are suggestions that epithelial cells might become sensitive to this chemical to result in a loss of epithelial integrity. We did not observe any change in paracellular permeability as measured by ^51^Cr-EDTA flux in adult *Hnf4α* colon null mice suggesting that spontaneous inflammatory disorder in this mouse model was not due to initial defects in tight junction dysfunction. In support of this, it was recently demonstrated that intestinal barrier loss in transgenic mice can accelerate the onset and severity of experimental colitis but is insufficient to lead in spontaneous inflammatory disease [29]. Our findings support the important conclusion that the solely loss of intestinal Hnf4α can initiate, by itself, epithelial defects that are resulting in spontaneous inflammatory disease.

A detailed gene profiling of *Hnf4α* colon null mice identified important classes of molecules with known functions in cell death, ion transport and cell cycle regulation. Our data indicate that three claudin family members (claudin-4, -8 and -15), crucial components of epithelial tight junctions, are deregulated long before the histopathological manifestations of the disease. *Hnf4α* colon null mice displayed a significant increase in the expression of claudin-4 and claudin-8 without any suggestion of relocalization in the mucosa, as studied by immunofluorescence. As expected, these modulations did not have an impact on mucosal permeability. Cldn15 was recently demonstrated to function as a paracellular ion transport without interfering in tight junction barrier function [23]. This function was confirmed in the mouse intestine by the generation of a claudin-15 knockout model that led to intestinal epithelial cell alterations, including enhanced proliferation and decreased electrical conductance without noticeable impact on mucosal permeability [24]. These findings are consistent with reduced claudin-15 expression in the upper half of the colonic mucosa in relation to the reduction of ion transport but not permeability that we observed in the *Hnf4α* colon null mice. There is growing evidence that specific defects in ion transport can lead to altered water movements and contribute to symptom generation and diarhea in IBD and spontaneous colitis in mice [30], [31]. Such defects can result in the alteration of acidic pH of the intestinal surface and favour bacterial adhesion to further facilitate microbial-epithelial interactions and translocation contributing to the inflammatory change. Functional variants of *SLC22A4* and *SLC22A5* ion transporter genes that alter their transcription and transporter functions are associated with the Crohn's disease *IBD5* locus [32]. Our observations are suggestive of a cascade effect of several ion transporters being altered in the *Hnf4α* colon null mice and to become important contributors to the long term onset of the disease in this context.

The detection of increased cleaved effector forms of caspase-3 as well as sporadic crypt hyperplasic regions in the colonic mucosa of *Hnf4α* mutant mice are consistent with the alterations predicted by the specific gene networks identified from the gene profiling analysis. Evidence is accumulating indicating that a defect in apoptosis is involved in the pathogenesis of IBD [33], [34]. In addition, E2F family members are recognized as important modulators of both cell division and apoptosis within developing tumors [35]. The long term observation of rare polyp initiation in some of the *Hnf4α* mutant mice could suggest that cumulated defects associated with colonic crypt distortion could eventually lead to tumor initiation under specific circumstances. Hnf4α down-regulates cell proliferation through the transcriptional activation of the cyclin-dependent-kinase inhibitor p21/CIP1/WAF1 [36]. However, the proliferation index of colonic epithelial cells in young adult mice was comparable between control and *Hnf4α* mutant mice (data not shown) and was consistent to what has been reported in the colonic embryonic situation [10]. Thus, the specific hyperplasic regions that develop in the absence of Hnf4α are probably the consequence of early molecular defects that influence the establishment of this phenotype. In accordance with this reasoning, IBD patients manifest an increased risk of developing colon cancer because persistent inflammation will lead to increased cellular turnover with selection pressures may result in the emergence of cells that are at high risk for malignant transformation [37].

We conclude that Hnf4α integrity is crucial to maintaining the fundamental properties of the colonic mucosa in protecting the host against luminal components that are likely to be disruptive, in the long term, to mucosal, immune homeostasis, potentially leading to episodes of colonic inflammation as observed in human IBD. *HNF4A* transcriptional regulator along with ion transport related target genes that include *CLDN15* represent promising candidates to dissect at the molecular level the delayed manifestation of IBD and might help to better define the predicting risk of IBD susceptibility among human populations.

## Supporting Information

Figure S1 Classification of modified genes in Hnf4α colon null mice by biological function
(0.35 MB XLS)

Table S1 Inflammatory mediator profile in diseased Hnf4α null colon
(0.02 MB XLS)

Table S2 Genes modulated by ≥2.0 in diseased Hnf4α null colon
(0.10 MB XLS)

Table S3 Junction forming genes in diseased Hnf4α null colon
(0.03 MB XLS)

Table S4 Ion transport associated genes
(0.04 MB XLS)
